# A qualitative study of the impact of the implementation of advanced access in primary healthcare on the working lives of general practice staff

**DOI:** 10.1186/1471-2296-6-39

**Published:** 2005-09-27

**Authors:** Sanjiv Ahluwalia, Maxine Offredy

**Affiliations:** 1 Watling Medical Centre, 108 Watling Avenue, HA8 0NR, London, UK; 2 Faculty of Health and Human Sciences, University of Hertfordshire, College Lane, Hatfield, AL10 9AB, UK

## Abstract

**Background:**

The North American model of 'advanced access' has been emulated by the National Primary Care Collaborative in the UK as a way of improving patients' access in primary care. The aim of this study was to explore the impact of the implementation of advanced access on the working lives of general practice staff.

**Methods:**

A qualitative study design, using semi-structured interviews, was conducted with 18 general practice staff: 6 GPs, 6 practice managers and 6 receptionists. Two neighbouring boroughs in southeast England were used as the study sites. NUD*IST computer software assisted in data management to identify concepts, categories and themes of the data. A framework approach was used to analyse the data.

**Results:**

Whilst practice managers and receptionists saw advanced access as having a positive effect on their working lives, the responses of general practitioners (GPs) were more ambivalent. Receptionists reported improvements in their working lives with a change in their role from gatekeepers for appointments to providing access to appointments, fewer confrontations with patients, and greater job satisfaction. Practice managers perceived reductions in work stress from fewer patient complaints, better use of time, and greater flexibility for contingency planning. GPs recognised benefits in terms of improved consultations, but had concerns about the impact on workload and continuity of care.

**Conclusion:**

AA has improved working conditions for receptionists, converting their perceived role from gatekeeper to access facilitator, and for practice managers as patients were more satisfied. GP responses were more ambivalent, as they experienced both positive and negative effects.

## Background

Patients' access to general practitioner (GP) appointments has been a key concern of the current British Labour government. Two national surveys [1,2] published in 1998 and 2000 highlighted the difficulty in accessing GPs, citing inconvenient surgery hours and long waiting. The government's response to this was to promise that "by the year 2004, patients will be able to see a primary care professional within 24 hours and a GP within 48 hours" [2]. Managing patients' requests for appointment has become increasingly important in general practice, as it is a national and local imperative, and features in the new GP contract in the United Kingdom (UK).

The National Primary Care Development Team (NPCDT) was set up to deliver the government's modernisation agenda in primary care by using the National Primary Care Collaborative (the Collaborative) to implement change. A key role of the Collaborative is to work with general practices and primary care trusts (PCTs) to help them modernise their services to better meet the needs of their patients. A priority for the Collaborative is improving access to primary care using the Advanced Access (AA) model, developed in the United States (US) [3-5]. Murray and Tantau's [5] solution for addressing the problem is borrowed from queuing theory and lean thinking, which are used in engineering and manufacturing, respectively. The underpinning principle is "doing today's work today". The solution is based on five principles, namely:

• understand the access demand on the practice

• clear the backlog of appointments

• review the appointment system

• develop contingency plans

• widen the mode of patient consultation.

The literature on AA focuses on: Improving access [5,6]; standards of access to quality in primary care [7,8]; drivers and barriers to implementing AA [9-11]; continuity of care versus quick access [12,13] and evaluation of AA [14]. Few of these articles provide empirical data. Those that provide empirical data demonstrate that AA improves access for patients to see GPs [14], AA adversely impacts on the ability of patients to see a GP of their own choice [13], and describes the barriers and drivers for implementing AA [11].

A wealth of literature suggests that the implementation and sustainability of innovations is dependent upon the perceptions of the users (patients and staff) of that innovation [15-17]. Moreover, the link between quality of working life and staff recruitment, retention, morale with ensuing effects on patient care is well recognised, and reflected in recent UK government policy [2,18]. There is, however, a real paucity of research into the impact of AA on the quality of working life amongst general practitioner staff. One questionnaire study explored the problems associated with working with the Collaborative and Advanced Access but to date there are no data on the effect of AA on other practice staff. Mays and Pope [19] suggest that qualitative methods are especially useful for understanding the perspectives of staff affected by health service reform. This research is therefore timely in its concentration on understanding the perceptions of receptionists, practice managers and GPs in relation to AA and its impact on their working lives.

### AIM

The purpose of this study was to explore staff's perspectives of the effect of introducing AA in their general practice on their workload and job satisfaction.

## Methods

### Settings

The study site was two boroughs situated in the south east of England with a resident population of approximately 207,000 and 306,000 people [20]. Both boroughs have ethnically and socio-economically diverse populations. The major causes of death in the boroughs are circulatory disorders and cancer. The six practices (table 1) in which the research was conducted were in different parts of the borough and represented different levels of deprivation and ethnic mix. Prior to the implementation of AA, the waiting time for a routine appointment in these practices was up to 10 days. After implementation the waiting time for an appointment to see a healthcare professional was less than 48 hours.

**Table 1:** Characteristics of the study's general practices

**Practice**	**No. of patients**	**Number of GPs**	**Full-time equivalent (fte) GPs**	**Patients per fte**	**No. of PMs**	**No. of practice nurses**	**Nurse roles**	**Number of receptionists**
A	11,700	6	6	1950	2	3	Chronic disease management	8 (part-time)
B	7,200	4	4	1800	1	2	Chronic disease, minor illness, telephone triage	3 (part-time)
C	6,800	2	2	3400	1	2	Chronic disease management	4 (part-time)
D	12000	7	5.5	2180	1	4	Chronic disease management	12 (part-time)
E	15000	9	7	2140	1	4	Chronic disease management, minor illness, telephone triage	14 (part-time)
F	10500	5	4.5	2330	2	3	Chronic disease management, minor illness, telephone triage	10 (part-time)

The practices varied in size from 7000 to 15000 patients. The numbers of doctors in the practices varied from 2 to 9. The number of patients per full time equivalent GP varied from 1800 to 3400. 5 practices were training practices. 4 practices had previously participated in research. For comparison, the average list size by weighted time equivalent for England was 1956 in 2003 [21] and median number of partners was between two and three [21].

### Ethical issues

Ethical approval was granted from the relevant research ethics committee covering the boroughs' two PCTs. Written consent was obtained from all participants prior to inclusion in the study.

### Participants

The selection of the six practices was purposive, reflecting the need for these practices to have implemented AA to meet the government's 48-hour access target. From all the practices invited to attend, one practice refused on the grounds that they had not implemented AA. None of the individual healthcare professionals approached for this study refused to participate. The researchers (SA & MO) attended practice meetings to inform the practice staff about the project and to seek their cooperation. GPs and practice managers with lead involvement in the implementation of AA in their practices were chosen for interview to ensure that they could share their experiences of advantages and disadvantages of previous appointment systems and AA. The receptionists on duty on the day of visit were asked to participate in the study. A total of 18 staff participated: 6 GPs, 6 practice managers and 6 receptionists. Information leaflets about the study were given to each participant.

### Interviews

**Figure 1:**
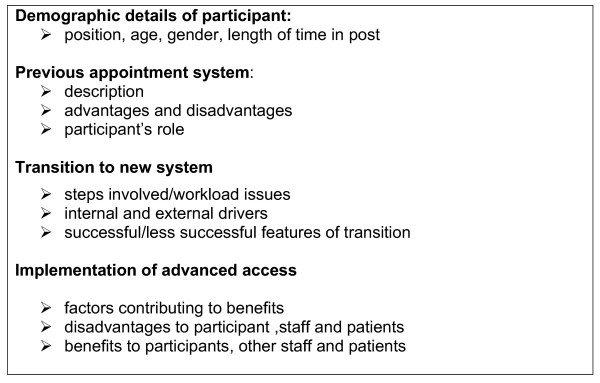
Interview schedule.

Semi-structured interviews were conducted with all participants, at a time and place convenient to them. The interviews were conducted informally and in a conversational style, which encouraged expansion of ideas. Each interview was tape-recorded and transcribed verbatim. The issues covered in the interviews are indicated in figure 1.

### Analysis

**Figure 2:**
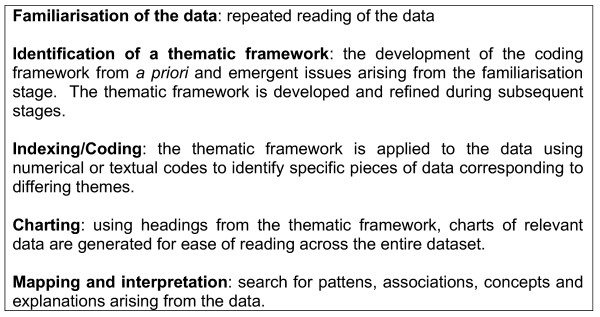
Data analysis stages.

Participants were given a number to protect their identity. Tape recordings generated by the 18 participants were transcribed verbatim using framework analysis [22] to identify concepts, categories and themes in the data. This analytic process provides systematic and visible stages of data analysis, broadly divided in to five stages shown in figure 2. It was developed in the context of applied policy research to meet specific information needs and provide outcomes or recommendations, often within a short timescale [23]. NUD*IST [24] software was used to manage the transcripts.

**Figure 3:**
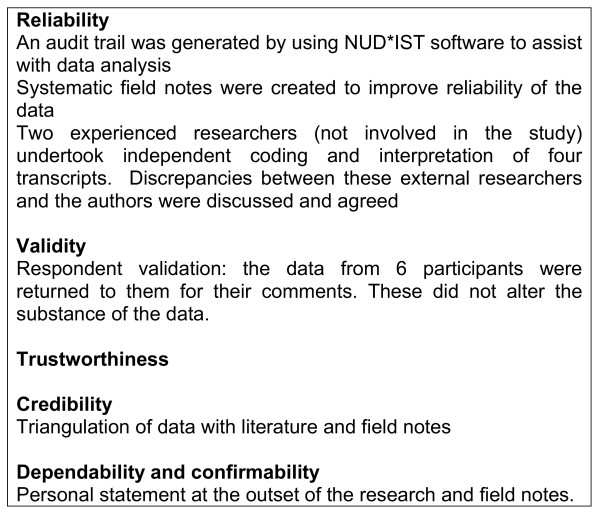
Approaches used to ensure the quality of the research.

Several approaches were used to ensure the quality of the research (figure 3). Validity was ensured by respondent validation, triangulation of data with literature and field notes. Generating an audit trail, keeping systematic field notes, and asking external researchers to code the data for a subset of the transcripts were ways of ensuring reliability. Where there were discrepancies these were discussed with the researchers. These methods of ensuring quality of research are consistent with established practice [25,26].

## Results

**Figure 4:**
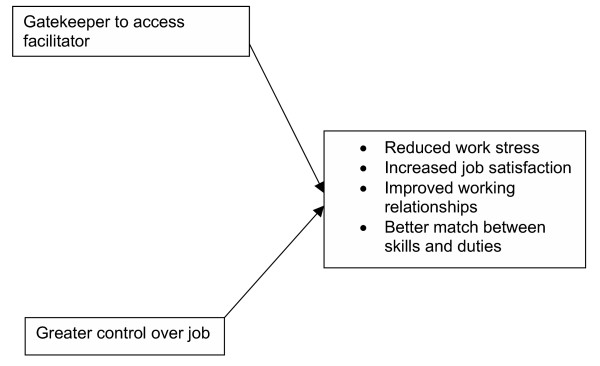
Themes for receptionists.

**Figure 5:**
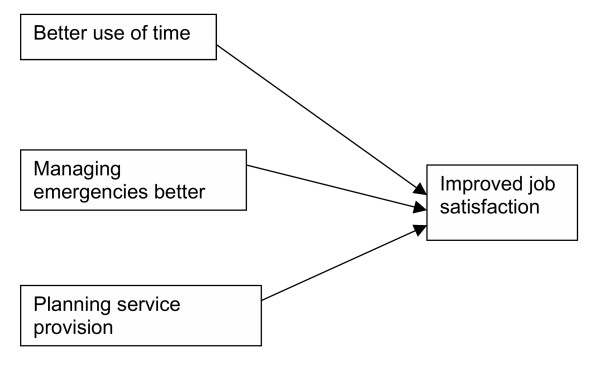
Themes for practice managers.

**Figure 6:**
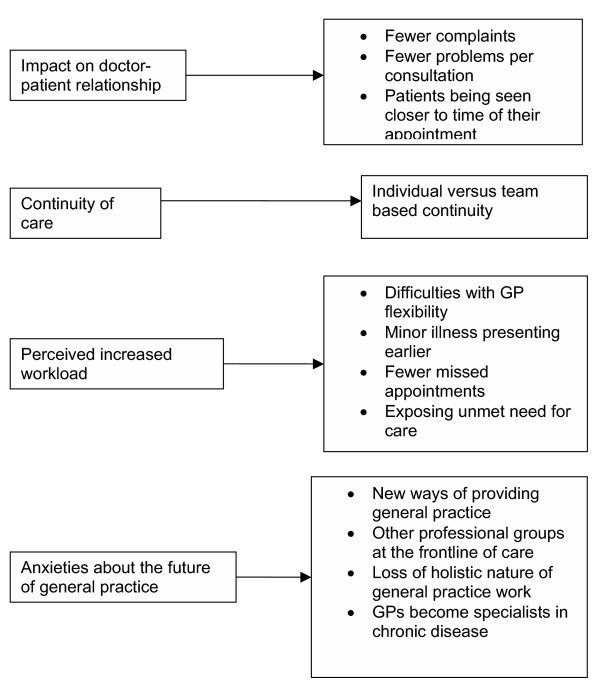
Themes for GPs.

The effects of AA are presented according to its impact on the working lives of receptionists (figure 4), practice managers (figure 5) and GPs (figure 6).

### Receptionists

#### From gatekeeper to access facilitator

Receptionists commented on changes in their levels of stress that took place with the introduction of AA. A significant problem for receptionists (R) under the previous appointment booking system was the stress and heightened tension encountered with patients, particularly when there was a shortage of appointments. This led to confrontations between receptionists and patients. Receptionists thus felt that patients perceived them as barriers or gatekeepers to being able to access GPs.

"It was very uncomfortable for receptionists to tell them about the waiting time because the patients saw it as our fault. We tended to get the blame; we took the brunt of it because when patients could not get to see the doctor of their choice and they did not want to wait 3–4 weeks because their problem is immediate, their aggravation and frustration would be taken out on us" (R2).

The way in which receptionists saw the change in their jobs with the introduction of AA was also enlightening. With the removal of the pressure of being seen as barriers to appointment, receptionists were able to adopt a role offering patients choice and access. They viewed their job as facilitating patients in being able to make appropriate appointments for their needs. The excerpt below underlines the point:

"With this system you can offer them (patients) an appointment on the day; it may not necessarily be the doctor of their choice though. You always finish the conversation with offering the patient an appointment. It is then their choice whether they take it or not... So I go off the phone feeling that I have offered them everything – three appointments – if it doesn't fit into their busy schedule then that is their choice. I am not denying them a health care professional" (R4).

#### Stress reduction through reduced confrontation with patients

Receptionists reported that the implementation of AA had reduced the level of stress of dealing with the public. With the implementation of AA long waits for an appointment to see a GP had disappeared. As a result, receptionists did not feel the need to have to triage patients, thereby being perceived as barriers or gatekeepers by patients. As a result, confrontations with patients had abated and receptionists were less stressed.

"For us receptionists, it has made our lives easier because patients phone up in the morning and they say can I have an appointment, and we say yes that's fine. The turnaround is much quicker; we are not on the phone for so long. We don't have to triage patients, which really is unacceptable. So it's made life much easier for us" (R5).

#### Improved sense of control over working day

Prior to the implementation of AA in their practices, receptionists would spend the majority of their time answering telephones, and would try and fit the rest of the job at quieter times. With the introduction of AA, receptionists found that demand for appointments was greatest early in the morning, which meant that they had a lot more time during the rest of the day, to complete their remaining duties, giving them a greater sense of control over their working day.

"But now you get the bulk of it (telephone calls) in the morning and then you get a set time of it in the afternoon, so it weighs itself out a little bit better.... Yes we can decide when to do scripts,(prescriptions) the rota and spend time showing the receptionists how to do things and sort their training out". (R4)

#### Greater job satisfaction

The shift in receptionists' views of their job from having to act as a barrier to GP appointments (prior to the introduction of AA) to becoming facilitators of choice for patients in provision of appointments (with the introduction of AA) and greater control over their working day increased their sense of job satisfaction as indicated by a receptionist:.

"It is so much better when you can offer the patient an appointment on the day; you come off the phone feeling satisfied rather than the hassle you used to have bargaining with them about how ill they are and why can't they come tomorrow" (R2).

#### Improved working relationships

Other benefits of implementing AA included improved working relationships as a consequence of feeling less stressed. Less confrontation at the reception desk with patients meant that receptionists felt they had a greater capacity to cope with the demands placed upon them by other members of the practice team. This led to better relationships between team members.

"I think the receptionists are far more relaxed and are relating to everyone else in a much more relaxed manner instead of being uptight because of patients. When someone (doctor or nurse) comes from a room the receptionists do not respond in such an uptight manner. It has definitely eased everything" (R3)

"Some of us are even smiling more, even with patients!" (R6)

### Practice managers

#### Better use of time

Prior to the introduction of AA, practice managers perceived high stress levels arising from the appointments system. The practice managers perceived several reasons for this stress. There was a high bureaucratic workload and time spent having to deal with complaints from patients as a result of poor access to GPs, pressure from having to cancel booked surgeries or find locums at short notice, and having to support stressed receptionists. The practice managers in this study reported that the introduction of AA improved their bureaucratic workload and use of time. They perceived a reduction in the numbers of complaints from patients. This had the effect of reducing paperwork and overall workload. It also meant that practice managers perceived patients were happier with the new appointments system.

"The advantages of the system to myself, is I don't get the complaints, it is a lot easier to organise, holidays with doctors, everyone is having a bit of a better life" (PM1).

#### Managing emergencies better

Coping with sudden illness or other unforeseen circumstances was easier and less stressful with AA than with the previous appointment booking system as exemplified below.

"As there are only a few pre-bookable appointments each session, it is much easier to rearrange appointments. Before this system (i.e. AA) came in, it was simply hell trying to cancel appointments because you know that you would be getting a lot of abuse from the patients" (PM4)

#### Planning service provision

Reductions in workload, stress from having to manage appointment crises at short notice, and reduced pressure from having to support staff meant that for the practice managers in the study were able to spend more time planning for the future.

"The advantage of the system is that I can plan in advance. I know exactly how many appointments we are going to have to offer each day and therefore I can plan if we need extra cover particularly for annual leave and training days." (PM5).

#### Improved job satisfaction

The overall impact of implementing AA meant practice managers perceived improvements for staff and patients. This in turn created a sense of job satisfaction for practice managers.

### General Practitioners

GPs in the study described the impact of implementing AA by its effect on the consultation between doctor and patient, fear of loss of autonomy, its impact on continuity of care, and anxieties about the future of general practice.

#### 1 Impact on the doctor patient consultation

The implementation of AA had reduced the time it took for a patient to be seen by a GP of their choice. As a result the GPs in this study perceived themselves as dealing with fewer complaints about long waits for an appointment or dealing with long problem lists (as patients were now better able to get an appointment quickly). They felt they were seeing patients nearer the time of their appointment and this improved the quality of the consultation.

#### 2 Increased workload for GPs

GPs perceived consultation rates as being higher with the additional burden of seeing more patients and therefore longer surgeries, as indicated below:

"..we find that because people are actually ringing in on the day when we run out of appointments, we feel we actually need to increase the number of appointments to accommodate them We often do that, and it just ends up with us seeing a lot more patients than we used to."(GP1)

Higher perceived consultation rates occurred for several reasons. These included fewer missed appointments with AA, improving access exposing unmet need for GP care, minor illness presenting earlier and mismatches between demand for appointments and supply of appointments.

#### 2a Difficulties with doctor flexibility

GPs understood the need for the appointments system to be closely related to the profile of demand for appointments from patients. However, this required doctors to be flexible with their time so that they did surgeries at times of high demand for appointments. However, such flexibility from doctors was limited. This was because of non-clinical commitments such as training, management, family and childcare issues.

"We (GPs) all try to be flexible; however, if there are partners who have got children and have other commitments, it is difficult for them to swap around (their sessions)" (GP2)

This meant that there was not enough doctor time at busy times to meet the demand for appointments requiring doctors to work longer sessions during these busy times.

#### 2b Earlier presentation of minor illness

Higher consultation rates also appeared to be partly related to earlier presentations of minor and self-limiting conditions. With the previous appointments systems long waits to get an appointment meant that patients with self limiting conditions would have got better by the due date of their appointment. With AA, this delay had disappeared, and therefore patients were being seen much sooner. This was seen as a source of frustration by GPs.

"We are going back to, 'I will just check before the weekend', or they will come in with a common cold and I will say "what would you like me to do for you? You have come with a cold". You feel they really need to see the Nurse Practitioner, not a GP."(GP2).

#### 3 Impact on continuity of care

The GPs in the study described the impact of AA on continuity of care. They described how AA affects continuity of care because patients were able to see a doctor or other healthcare professional other than their registered doctor; and there were difficulties gaining an appointment with a doctor of the patient's choice.

The idea of a single GP providing continuous care appeared to be changing. As a consequence, GPs found that they needed to re-assess patients every time, which they regarded as stressful and labour-intensive.

"She (the patient) doesn't really care whom she sees, and she doesn't perceive it as being important who she sees. But then there's tremendous work for doctors, picking up each other's work. The doctors are used to seeing the same patients coming back to them so they know where they are, and they know where they stand. Often you are starting a fresh all the time with patients we don't always know" (GP2).

Other GPs replaced the idea of continuous care by a single GP with continuous care provided by a team of practitioners.

"If there is somebody with an acute illness that may be related to their chronic illness, does it matter that they see somebody else? It only matters if you don't keep good clinical records and if you don't communicate with your partners." (GP1).

A combination of increased perceived workload and the erosion of continuity of care meant that GPs feared a loss of autonomy over their working lives.

#### 4 Anxiety about the future of general practice

The perceived increase in minor illness and workload meant that practices had to consider alternative ways of providing healthcare. Various options were considered by the practices studied. These included the use of telephone triage, self-help and education material for patients, nurse telephone advice and triage, pharmacist and nurse consultations for minor illness, and providing consultations by alternative means such as emails.

"We have a very successful nurse advice line which the doctors do use to some extent to follow up a patient. However, we really do need more time for that. So we are looking to develop the advice line in the afternoon and that the nurse is available to do triage in the afternoons."(PM2)

These alternatives to GP-led healthcare suggested that in the long term GPs would stop being the frontline providers of healthcare. Such a role would be taken over by nurses and other professional groups. GPs expressed a fear that the holistic nature of providing care in the future would be replaced by a system where doctors became specialists caring for chronic disease.

"The nature of general practice will change because the patient will not have continuity of care. They will not come to you for their minor bits and pieces (as with the previous appointments system), they will only come for the major things, and GPs will become consultants in general practices. I think that is probably the way it is going to go. It will change the exclusive character of British general practice, which I think will be sad." (GP3).

With the implementation of AA, GPs perceived doctors popular with patients being busier than GPs less popular. GPs were seeing a greater variety of patients. However, because the demand for appointments was greater than the supply of appointments, many patients with long term relationships were being seen by other GPs and healthcare professionals. In some cases it has encouraged better use of the whole team whilst maintaining flexibility for doctors to be able to see whoever they felt was appropriate.

"It has meant for me that some of my hangers-on are prepared to see other people, so we are using the whole team and they are beginning to build relationships with the other members of the team, which is really positive for me. I don't feel bereaved over the loss of these patients." (GP4).

## Discussion

### Summary of main findings

This paper has elucidated some of the advantages and disadvantages to general practice staff arising from the implementation of AA in their practices. Whilst practice managers and receptionists see AA as having a positive effect on their working lives, GPs had a more ambivalent reaction.

For GPs in this study, the perception of stress from patient contact has reduced because patients have complained less about poor access; they now attend the consultation with fewer, more trivial complaints. Other benefits included better time management and better use of the whole team, particularly for doctors perceived as being popular with patients. Concerns included a perception of increased workload, related to fewer non-attendances by patients, earlier presentation of minor illness and difficulties in getting doctors to work more closely to the profile of demand from patients causing a mismatch between demand for appointments and supply. Other concerns were the effect of AA on continuity of care, and the impact of AA on the future of GP work. These concerns seemed to reduce job satisfaction and engendered a fear of loss of autonomy.

### Implications of this study and relationship to other work

#### Higher perceived workload

This study suggested that GPs perceive their workload as having increased with the advent of AA. This is consistent with the British Medical Association's survey of GP workload in 2001 [27], and Pickin et al's [14] study on AA in UK general practice. It is unclear from this study whether AA has fuelled the increase in workload and demand for appointments, or whether AA has permitted the unmasking of previously unmet healthcare related needs. It is also unclear what the consequences are from a resource perspective with increasing workloads.

AA has had the perceived impact of increasing GP workload, increasing patient demand for appointments, and changing the way care is provided by GPs so that the greater variability in case-mix along with fragmentation of care become sources of increased stress and reduced job satisfaction. Gosden et al [28] have shown that major work-related stressors for GPs include increasing patient demand, and increasing workloads. Similarly, less control over how care is provided is a significant factor in reducing job satisfaction.

#### Continuity of care

Some GPs, in this study, defined continuity of care as being provided by individual GPs. These doctors felt that AA had eroded the personal relationship between doctor and patient. By contrast, other GPs defined continuity of care as being provided by groups of healthcare professionals. These GPs did not see AA as adversely affecting continuity of care. Windridge et al [13], in their qualitative patient study, highlighted that AA adversely impacted on the ability of patients to see a GP of their choice. Yet, doctors and patients regard continuity of care as being important [29]. This contrasts with government policy to increase interprofessional working, and offer patients greater choice in accessing healthcare [30-33].

#### Impact on practice managers

The beneficial effects of AA in reducing work-related stress for practice managers are particularly welcome in view of the previously identified causes of psychological morbidity in this group [34].

### Strengths and limitations of the study

The study is the first of its kind to provide qualitative insights into the working lives of GPs, practice managers and receptionists following the introduction of AA in their practices. It highlights that the effects on GPs remain unclear and there is need for further research in this area. The practices selected for this study were diverse and not dissimilar to the profile of English general practices overall. The interviewers were independent and not perceived as having a vested interest. Outside researchers validated the analytical framework. However, its limitation is that the six practices used in the study may not be representative of the practices that have implemented AA because they could be seen as early adopters of change, even though they included ethnically and socio-economically diverse populations. It is possible that these practices were influenced by the same PCT policies and PCT access facilitator, thereby reducing the potential generalisability of such a study. The focus on the views of doctors who had a lead involvement in introducing AA may have introduced perceptions more favourable than otherwise.

## Conclusion

AA has improved the working conditions for receptionists, converting their perceived role from gatekeeper to facilitator, and practice managers were also more satisfied with their jobs. GP responses were more ambivalent, as they experienced both positive and negative effects.

## Competing interests

The author(s) declare that they have no competing interests.

## Authors' contributions

SA and MO designed the study, carried out the analyses, and contributed equally to the writing of this article.

## Pre-publication history

The pre-publication history for this paper can be accessed here:


